# Imaging flow cytometry-based multiplex FISH for three *IGH* translocations in multiple myeloma

**DOI:** 10.1038/s10038-023-01136-2

**Published:** 2023-03-08

**Authors:** Taku Tsukamoto, Masaki Kinoshita, Kazuhiro Yamada, Hodaka Ito, Toshikazu Yamaguchi, Yoshiaki Chinen, Shinsuke Mizutani, Takahiro Fujino, Tsutomu Kobayashi, Yuji Shimura, Johji Inazawa, Junya Kuroda

**Affiliations:** 1 Division of Hematology & Oncology, Department of Medicine, Kyoto Prefectural University of Medicine, Kyoto, Japan; 2 Sysmex Corporation, Hyogo, Japan; 3 General Laboratory, Bio Medical Laboratories, Inc., Tokyo, Japan; 4 Department of Blood Transfusion and Cell Therapy, Kyoto Prefectural University of Medicine, Kyoto, Japan; 5 Department of Molecular Cytogenetics, Medical Research Institute, Tokyo Medical and Dental University, Tokyo, Japan; 6 Research Core Center, Tokyo Medical and Dental University, Tokyo, Japan

**Keywords:** Cytogenetics, Myeloma

## Abstract

Three types of chromosomal translocations, t(4;14)(p16;q32), t(14;16)(q32;q23), and t(11;14)(q13;q32), are associated with prognosis and the decision making of therapeutic strategy for multiple myeloma (MM). In this study, we developed a new diagnostic modality of the multiplex FISH in immunophenotyped cells in suspension (Immunophenotyped-Suspension-Multiplex (ISM)-FISH). For the ISM-FISH, we first subject cells in suspension to the immunostaining by anti-CD138 antibody and, then, to the hybridization with four different FISH probes for genes of *IGH*, *FGFR3*, *MAF*, and *CCND1* tagged by different fluorescence in suspension. Then, cells are analyzed by the imaging flow cytometry MI-1000 combined with the FISH spot counting tool. By this system of the ISM-FISH, we can simultaneously examine the three chromosomal translocations, i.e, t(4;14), t(14;16), and t(11;14), in CD138-positive tumor cells in more than 2.5 × 10^4^ nucleated cells with the sensitivity at least up to 1%, possibly up to 0.1%. The experiments on bone marrow nucleated cells (BMNCs) from 70 patients with MM or monoclonal gammopathy of undetermined significance demonstrated the promising qualitative diagnostic ability in detecting t(11;14), t(4;14), and t(14;16) of our ISM-FISH, which was more sensitive compared with standard double-color (DC) FISH examining 200 interphase cells with its best sensitivity up to 1.0%. Moreover, the ISM-FISH showed a positive concordance of 96.6% and negative concordance of 98.8% with standard DC-FISH examining 1000 interphase cells. In conclusion, the ISM-FISH is a rapid and reliable diagnostic tool for the simultaneous examination of three critically important *IGH* translocations, which may promote risk-adapted individualized therapy in MM.

## Introduction

Multiple myeloma (MM) is the second most frequent hematologic malignancy which is cytogenetically and molecularly highly heterogeneous among patients [1–6]. The identification of cytogenetic abnormality is essential in the clinical practice of MM. Especially, the detection of structural abnormalities of chromosomal translocations, such as t(4;14)(p16;q32) for hybrid gene fusions between *IGH* and *FGFR3* or *MMSET*, t(14;16)(q32;q23) for *IGH*/*MAF* fusion gene, and t(11;14)(q13;q32) for *IGH*/*CCND1* fusion gene, and various types of numerical abnormalities, including 1q gain/amplification, and deletion 17p, is indispensable for the prediction of treatment response and prognosis and the choice of therapeutic strategy. Epidemiologically, t(4;14), t(14;16) and t(11;14) are present in approximately 10–25, 3–7, and 15–20% of patients with newly diagnosed MM (NDMM), respectively [1, 2, 5, 7]. Since the acquisition of these chromosomal translocations has been considered the initial founding step in the development of myeloma-initiating cells, all myeloma cells share the same translocation in each patient, while these three major structural *IGH* translocations are generally mutually exclusive. Importantly, these three translocations also strongly associate with the profiles of co-existing genetic/molecular abnormalities and gene expression patterns of myeloma cells [8, 9], and have significant impacts on the clinical features, the efficacy of treatment strategies, and the eventual prognosis of patients in clinical practice. Indeed, the presence of t(4;14) or t(14;16) is incorporated as a component of poor prognostic factors in the revised International Staging System [10], while the prognostic impact of t(11;14) has been controversial in association with various confounding factors, such as the type of treatment and the co-existing additional chromosomal abnormalities [11–13]. However, the prognosis of patients with t(4;14) or t(14;16) has been improved by the therapeutic approaches incorporating proteasome inhibitors or monoclonal antibodies against CD38 or SLAMF7 [14–17], but not by high-dose melphalan supported by autologous stem cell transplantation or by immunomodulatory drugs. The efficacy of BCL2 inhibitor venetoclax is particularly prominent in patients with t(11;14), while not in patients without t(11;14) [11, 13]. Thus, the judgment of the presence or the absence of these three translocations is the prerequisite for the treatment selection and the prediction of prognosis in the clinical practice of MM.

The traditional Giemsa (G)-banding technique has several shortcomings, as it requires the presence of metaphase spreads of fresh living tumor cells, but not frozen cells, and has the difficulty in analyzing low proliferative cells, such as myeloma cells. In addition, G-banding is not a high-resolution technique and is insufficient for the detection of t(4;14) owing to its involvement in subtle telomeric regions [7]. To overcome these, FISH for the gene of interest has been widely applied for chromosomal diagnosis in MM. Due to its primary role in mapping genes on chromosomes not only in metaphase cells but also in interphase cells, double-color (DC) interphase-FISH offers a practical advantage in detecting gene fusion by chromosomal translocation even in low proliferative myeloma cells [18]. However, with the conventional DC-FISH on fixed whole bone marrow (BM) mononucleated cells attached to the glass slide, there is a need for repeating the direct observation of more than hundreds of cells (usually 200–400 cells) probed by DC-FISH probes for different types of translocations individually under a fluorescence microscope. Even with the enrichment of CD138-positive cells, cell sorting procedures are time and cost-consuming, and the situation is the same in that investigators need to repeat the direct observation for different types of translocations individually.

The environment of clinical practice and laboratory tests varies widely among countries and institutes. To make a cytogenetic diagnosis of myeloma cells more convenient in daily practice universally, we in this study developed a new diagnostic modality of the multiplex FISH in immunophenotyped cells in suspension (Immunophenotyped-Suspension-Multiplex (ISM)-FISH), using the imaging flow cytometry which can simultaneously examine three disease-specific chromosomal translocations, i.e., t(11;14), t(4;14) and t(14;16) in CD138-positive tumor cells of plasma cell dyscrasia, including MM and monoclonal gammopathy of undetermined significance (MGUS).

## Materials and methods

### Cell lines and patient-derived samples

Human myeloma-derived cell lines (HMCLs), KMS-11, KMS-21-BM, KMS-26, and acute myelogenous leukemia-derived cell line HL-60 were purchased from the Japanese Collection of Research Bioresources (Osaka, Japan). BM samples were obtained from patients with MGUS (*n* = 12), NDMM (*n* = 23), and RRMM (*n* = 35) between September 2017 and March 2021 at the Division of Hematology and Oncology, Department of Medicine, Kyoto Prefectural University of Medicine (KPUM). MGUS/MM was diagnosed based on the International Myeloma Working Group 2014 criteria [19]. Written informed consent was obtained from all patients. The study was conducted in compliance with the Declaration of Helsinki, and the study protocol was approved by the institutional review board of KPUM (ERB-C-930-1).

### Immunophenotyped-suspension-multiplex (ISM)-FISH using the imaging flow cytometry

**Fig. 1:**
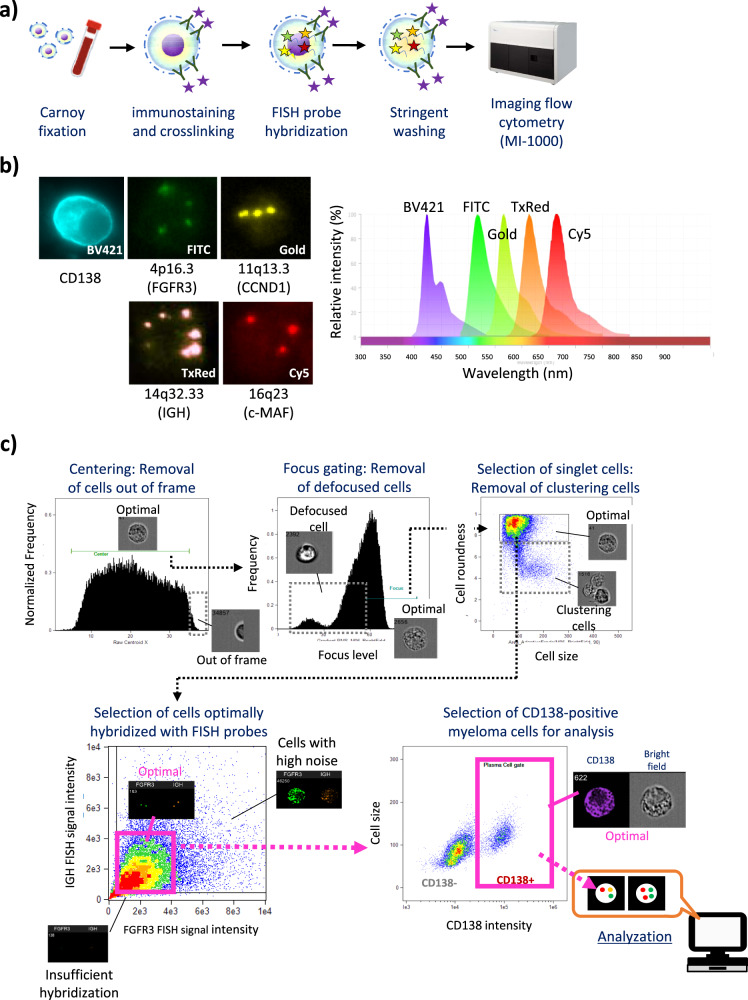
Procedure of the immunophenotyped-suspension-multiplex fluorescence in situ hybridization (ISM-FISH). **a** The brief flow diagram of ISM-FISH. **b** Fluorescence utilized for ISM-FISH included BV421 conjugated to an anti-CD138 antibody, and fluorescein isothiocyanate (FITC), Gold, Texas Red (TxRed), and Cy5 to FISH probes for *FGFR3*, *CCND1*, *IGH*, and *MAF*, respectively. **c** The flow diagram of the imaging analysis for the detection of FISH signals in CD138-positive myeloma cells using imaging flow cytometry

In brief, ISM-FISH was performed as shown in Fig. 1a. BM fluid was subjected to hypotonic treatment with 75 mM KCL. Then, BM nucleated cells (BMNCs) were fixed in Carnoy’s solution (3:1, methanol; acetic acid). Fixed cells were washed with 1x PBS containing 0.5% BSA (Proliant Biologicals, Ankeny, IA, USA) twice, and were resuspended in 1x PBS containing 0.2% Pluronic F (PF)-127 (Sigma Aldrich, St. Luis, MO), and were stained by Brilliant violet (BV) 421-conjugated anti-human CD138 antibody (clone MI15) (BioLegend, San Diego, CA, USA) diluted at 1:20 with 2 mM bissulfosuccinimidyl suberate (BS3) crosslinking (Thermo Fisher Scientific, Waltham, MA, USA) (Fig. 1b). The immunostaining reaction was stopped by the addition of 1 x TBS containing 0.2% PF-127, and cells were washed and resuspended in 1 x PBS containing 0.2% PF-127. Four customized FISH probes for Texas Red (TxRed)-conjugated probe for *IGH*, FITC-conjugated probe for *FGFR3*, Gold-conjugated probe for *CCND1*, and Cy5-conjugated probe for *MAF* (Cytocell, Cambridge, UK) Fig. 1b were prewarmed at 37 °C, mixed with cells, and were subjected to denature at 92 °C for 5 min, followed by hybridization at 42 °C for at least 16 h. Hybridized cells were resuspended with 2 × SSC buffer containing 0.2% PF-127, were washed, resuspended with 0.4 × SCC containing 0.2% PF-127 prewarmed, and were incubated for 2 min at 73 °C. Then, more than 2.5 × 10^4^ cells per sample were subjected to the imaging flow cytometric analysis using MI-1000 (Sysmex, Hyogo, Japan). Scatter plot analysis was performed with IDEAS software (ver. 6.2) (Amnis, Seattle, WA, USA). In the process of imaging flow cytometric analysis, we first isolated singlet cells that were optimal for the investigation by removing cells that were out of frame, defocused cells, and clustering cells under bright field observation. Next, we selected cells optimal for cytogenetic analysis by removing cells that were insufficiently hybridized with FISH probes or cells with high noise, and, then, sorted CD138-positive cell fraction for evaluating the cytogenetic status of myeloma cells using the FISH spot counting tool (Sysmex) (Fig. 1c).

### Standard double-color (DC)-FISH

Conventional standard DC-FISH for *IGH*/*CCND1*, *IGH*/*FGFR3*, and *IGH*/*MAF* were separately performed for each sample as described previously [18, 20]. Probes utilized for standard DC-FISH were Vysis LSI *IGH*/*FGFR3* Dual Color Dual Fusion Probes, Vysis LSI *IGH*/*CCND1* Dual Color Dual Fusion Probe, and Vysis LSI *IGH*/*MAF* Dual Color Dual Fusion Probes (Abbott, Abbott Park, IL). An independent analysis was routinely performed on 200 interphase cells for three chromosome translocations, and on 1000 interphase cells in case needed. The cut-off values of detection for three translocations were 1.0%.

### Statistics

Statistical analyses were performed with EZR, a graphical user interface for R version 4.1.1. (The R Foundation for Statistical Computing, Vienna, Austria) [21]. The student’s t-test was used to compare continuous variables between groups, and a p-value less than 0.05 was considered significant.

## Results

### ISM-FISH enables the simultaneous evaluation of three chromosomal translocations in myeloma cells

**Fig. 2:**
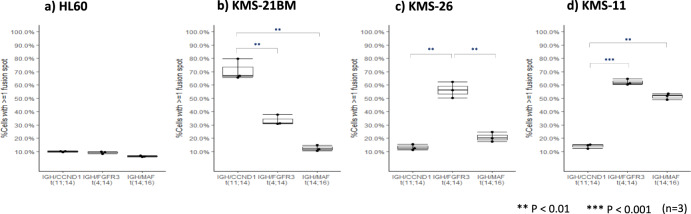
Validation of the diagnostic potency of ISM-FISH. Three chromosomal translocations of t(11;14), t(4;14), and t(14;16) were simultaneously examined in (**a**) HL-60 cells (negative control). **b** KMS-21BM cells, (**c**) KMS-26 cells, and (**d**) KMS-11 cells. Y-axis represents the ratio of cells with more than 1 fusion signal. **P < 0.01; ***P < 0.001

First, we investigated whether our ISM-FISH system enables the simultaneous evaluation of the presence and/or absence of the three target chromosomal translocations, i.e., t(11;14) for *IGH*/*CCND1*, t(4;14) for *IGH*/*FGFR3*, and t(14;16) for *IGH*/*MAF*. For this purpose, we utilized three HMCLs, KMS-21BM cells harboring t(11;14), KMS-26 cells harboring t(4;14), and KMS-11 cells with concomitant two chromosomal translocations of t(4;14) and t(14;16) [22, 23]. As a negative control, we utilized HL-60 cells without any of the three translocations. As shown in Fig. 2a, the examinations on HL-60 cells revealed that our system produced false-positive signals for three chromosomal translocations in approximately 10% of the cells examined. In the three HMCLs, our system identified the presence of the chromosomal translocation(s) that should be present in each HMCL, while also showing both false-negative signals in 20.2 to 51.1% (median: 39.2%) cells and false-positive signals in 10.4 to 37.8% (median: 15.0%) cells (Fig. 2b–d). However, the differences between the rates for true-positive signal(s) and false-positive signals were statistically significant in all three HMCLs examined. These indicate the qualitative diagnostic ability of our system, while also the need for the establishment of an optimal threshold for a false-positive signal for each chromosomal translocation.

### Sensitivity of ISM-FISH for the simultaneous evaluation of three chromosomal translocations

**Fig. 3:**
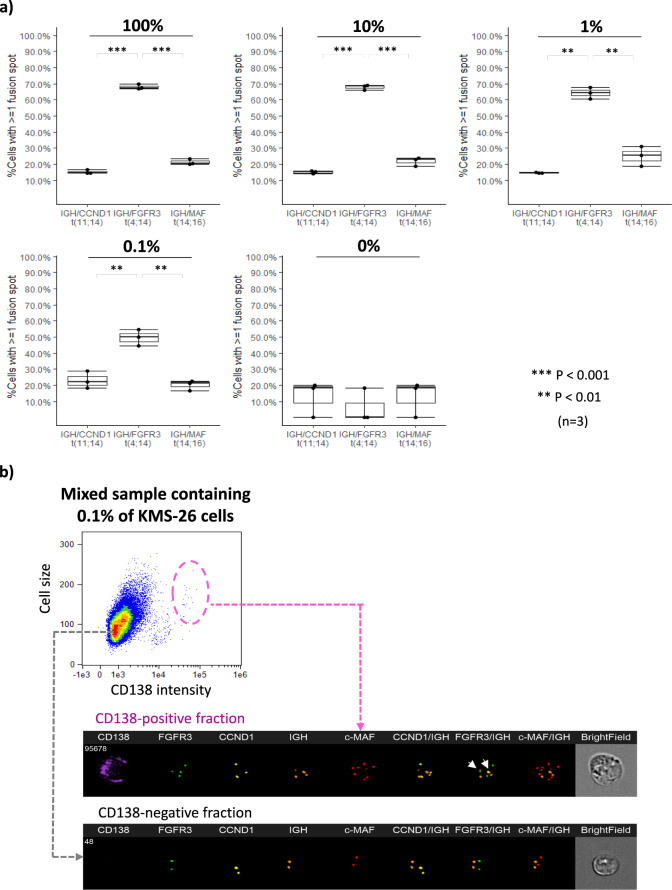
Sensitivity of ISM-FISH in HMCL. **a** KMS-26 cells possessing *IGH*/*FGFR3* translocation were mixed with HL-60 cells by different ratios (100%, 10%, 1%, 0.1%, 0%), and were subjected to the ISM-FISH system. Y-axis represents the ratio of cells with more than 1 fusion signal. **P < 0.01; ***P < 0.001. **b** The representative view of ISM-FISH examining a mixed sample containing 0.1% of KMS-26 cells and 99.9% of HL-60 cells. Arrows indicate the fusion signal of *IGH*/*FGFR3*

We next investigated the sensitivity of the ISM-FISH system. For this purpose, we mixed KMS-26 cells harboring *IGH*/*FGFR3* translocation and HL-60 cells by different ratios (100%, 10%, 1%, 0.1%, 0%) and subjected mixed samples to the ISM-FISH system. As shown in Fig. 3, %cells with ≥1 fusion spot for *IGH/FGFR3* showed almost the same value around 60–70% when KMS-26 cells are mixed at 1–100%. Even at 0.1% of KMS-26 cells, the ISM-FISH system detected a significantly higher proportion of cells with *IGH/FGFR3* translocation (true-positive signals) compared with false-positive signals for *IGH/CCND1* and *IGH/MAF* in the presence of 0.1% of KMS-26 cells with the background of 99.9% of HL-60 cells, suggesting the sensitivity of ISM-FISH is at least 1%, and possibly up to 0.1%. Additional experiments showed that the system was not sensitive in case translocation-positive cells were less than 0.1% (data not shown).

### ISM-FISH for patient-derived BM samples containing various proportions of myeloma cells

**Fig. 4:**
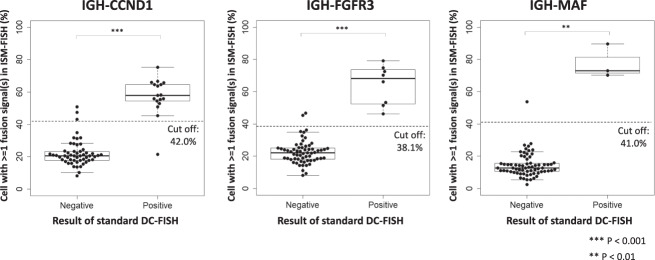
Results of ISM-FISH and standard DC-FISH in patient-derived BMNCs. Y-axis represents the ratio of cells with more than 1 fusion signal in ISM-FISH. Dash lines represent the cut-off between positive and negative results. **P < 0.01; ***P < 0.001

**Fig. 5:**
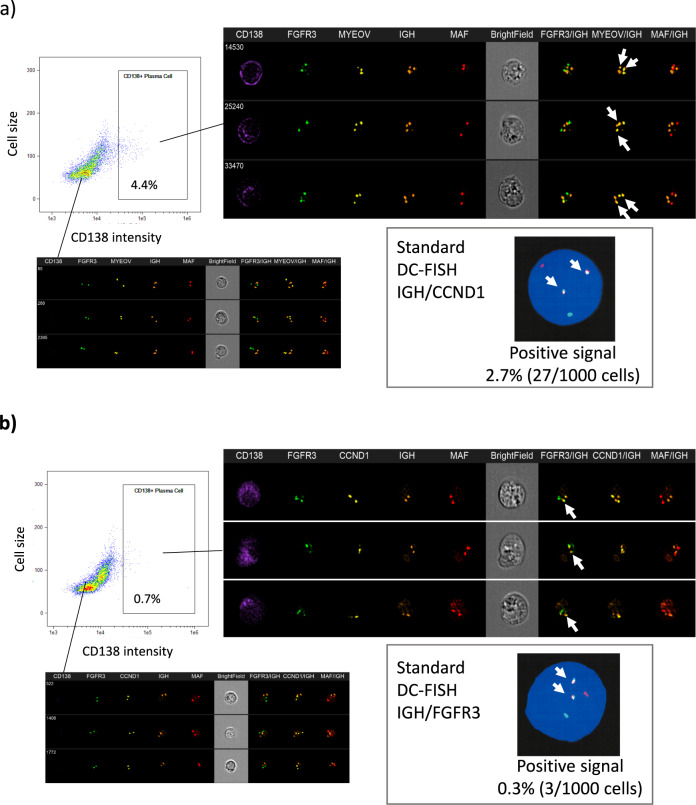
Representative results of two patients’ standard DC-FISH-negative (200 cells)/ISM-FISH-positive samples. **a** A case (No.41 in Table S1) of MGUS whose BMNCs contained 4.4% of plasma cells counted in BM aspirate smear. While the result of standard DC-FISH for *IGH*/*CCND1* examining 200 BMNCs was negative, standard DC-FISH examining 1,000 BMNCs detected fusion signals of *IGH*/*CCND1* in 2.7% of BMNCs. ISM-FISH revealed the presence of an *IGH*/*CCND1* fusion signal (arrow) in CD138-positive plasma cells in this case. **b** A patient with RRMM (No.33 in Table S1) whose BMNCs contained 2.0% of myeloma cells in BM aspirate smear. While standard DC-FISH examining 200 BMNCs showed negative results, DC-FISH examining 1000 BMNCs detected fusion signals of *IGH*/*FGFR3* in 0.3% of BMNCs. ISM-FISH detected the cells harboring the *IGH*/*FGFR3* fusion gene (arrow)

Finally, we examined the clinical utility of ISM-FISH for the simultaneous evaluation of three chromosomal translocations in comparison with standard DC-FISH in 70 patient-derived BM samples containing various proportions of tumor cells Table S1. In ISM-FISH, the negative cut-off threshold was determined by mean + 3 standard deviation (%) of cells with more than 1 fusion spot(s) of 30 randomly selected translocation-negative patients with standard FISH. Accordingly, the negative cut-off thresholds (%) for *IGH*/*CCND1*, *IGH*/*FGFR3*, and *IGH*/*MAF* were determined to be 42.0%, 38.1%, and 41.0%, respectively (Fig. 4). With these settings, *IGH*/*CCND1* translocation was considered positive in 16 patients by both standard DC-FISH (200 cells) and by ISM-FISH, in 3 patients only by ISM-FISH, and in one patient only by standard DC-FISH (200 cells). In 3 ISM-FISH-positive/standard DC-FISH (200 cells)-negative samples, the extensive observation of 1,000 interphase cells with standard DC-FISH revealed the presence of a small proportion of *IGH*/*CCND1*-positive cells (1.2% (data not shown) and 2.7% (Fig. 5a) in two samples, while did not detect positive cell in one sample. *IGH*/*FGFR3* translocation was positive in 8 patients by standard FISH, while in two more samples by ISM-FISH. The extensive observation of 1,000 interphase cells with standard DC-FISH revealed the presence of a small proportion of *IGH*/*FGFR3*-positive cells in two samples (3.4% (data not shown) and 0.3% (Fig. 5b). As for *IGH*/*MAF*, while 3 samples were considered positive by standard DC-FISH, one more sample was also considered to be positive by ISM-FISH (Table 1, Fig. 4). In one sample which was ISM-FISH-positive/standard DC-FISH (200 cells)-negative for *IGH/MAF*, the extensive observation of 1,000 interphase cells with standard DC-FISH did not detect *IGH*/*MAF*-positive cells (data not shown). Collectively, ISM-FISH showed a positive concordance of 96.6% and a negative concordance of 98.8% with standard FISH (1,000 cells). ISM-FISH was found to be more sensitive compared to standard DC-FISH (200 cells).

**Table 1:** Results of standard double-color (DC)-FISH and the immunophenotyped-Suspension-multiplex (ISM)-FISH in patient-derived bone marrow nucleated cells

	Total *n* = 70	ISM-FISH	
	Standard DC-FISH	Positive, *n*	Negative, *n*
t(11;14) for *IGH*/*CCND1*	Positive, *n*	16	1
	Negative, *n*	3	50
t(4;14) for *IGH*/*FGFR3*	Positive, *n*	8	0
	Negative, *n*	2	60
t(14;16) for *IGH*/*MAF*	Positive, *n*	3	0
	Negative *n*	1	66

## Discussion

Various attempts have been made to use flow cytometry technology to evaluate chromosome status since the development of flow karyotyping in the mid-1970s [24, 25]. Then, the combination assay of the FISH technique and the immunophenotyping of cells of interest using flow cytometric procedure, the so-called immune-S-FISH, has been introduced as a successor modality [25–29]. Unlike the standard FISH for cells attached to the glass slide, the immuno-S-FISH enabled the high-throughput evaluation of the chromosome status of many immunophenotyped cells in suspension. In addition, the automated analysis with digital image capture enables the standardized evaluation of chromosomal status which may diminish operator bias. As the result, the immune-S-FISH technique has been successfully translated into the use for the detection of diagnostically/prognostically important chromosomal abnormalities in hematologic malignancies [27–31]. However, in those previous studies, the immune-S-FISH technique has been only applied for the simultaneous detection of up to two types of chromosome abnormalities in hematologic malignancies, such as trisomy 12 and del(17p) in chronic lymphocytic leukemia, or t(15;17) in acute promyelocytic leukemia [28, 29]. Therefore, the ISM-FISH presented in this study is the first which enables the simultaneous investigation of three chromosomal translocations by using five different fluorescence for four genomic regions and one cell surface antigen. This unique property is especially useful in distinguishing disease subtypes of the same disease entity defined by the type of chromosomal abnormality. Potential target candidate diseases for the application of ISM-FISH other than MM include B cell lymphomas consisting of various subtypes, including double/triple hit lymphoma, defined by specific and/or prognostically important *IGH* chromosomal translocations, such as those involving *BCL2*, *MYC*, *BCL6*, or *CCND1*, or acute leukemias with subtype-specific chromosomal translocations involving core binding factors or Philadelphia chromosome [31–34]. This technique will be also useful for evaluating /monitoring the presence of simultaneous hematologic diseases e.g., BM involvement of myeloma, lymphoma, CLL, and myelodysplastic syndrome.

The optimal selection of biologically/pathologically specific antigens is crucial for the accurate immunophenotyping of cells of interest in the ISM-FISH. Among various antigens expressed on myeloma cells, we selected CD138 as the marker antigen in this study, as CD138 is widely expressed in the plasma cells of most patients with MGUS and MM [35, 36]. The use of CD138 as the selection marker makes the sensitivity of our system at least up to 1%, which is much higher than the sensitivity with standard DC-FISH, and the high sensitivity with the ISM-FISH is particularly advantageous in the setting of clinical practice for the chromosomal diagnosis in MGUS and MM. While the plasma cell ratio in BM is defined to be below 10% for the diagnosis of MGUS which is a pre-malignant phase of MM, it is frequently around 0–3%. Among various factors proposed as risk factors for the progression to MM which occurs in approximately 10% of patients with MGUS, clonal expansion of chromosomal translocation-positive cells has been suggested to be one of the predictors for disease progression [37–39], while the proportion of clonal plasma cells in BMNCs is not infrequently sufficient to be analyzed by the standard DC-FISH in MGUS [38]. Thus, our system of ISM-FISH is particularly useful for the investigation of chromosomal translocations in MGUS compared to standard DC-FISH. Even with untreated MM at diagnosis, the proportion of myeloma cells in BMNCs obtained by BM aspiration is occasionally low, sometimes less than 5% due to the patchy and heterogenous intra-BM distribution/infiltration of myeloma cells in BM [40]. In such a situation, it is expectable that the ISM-FISH may overcome the difficulty of cytogenetic diagnosis by standard DC-FISH due to the low percentage of myeloma cells in BM fluid.

The technical limitation of the ISM-FISH developed here was a relatively high ratio of both false-positive and false-negative fusion signals in an individual sample. The false-positive fusion signal of different fluorescent signals for different target genes that are spatially distinct hybridization spots occurs due to the superimposed spots on two-dimensional (2D) projection of three-dimensional (3D) cells which occurs not only in ISM-FISH but also in the standard DC-FISH; however, this formidable problem could be further enhanced with the ISM-FISH for examining four genes compared to the standard DC-FISH examining two genes, because the simultaneous hybridization of more target genes increases the frequency of incidental signal overlap. In addition, the use of unattached spherical moving cells in solution may also increase the incidental overlap of different fluorescent signals in the imaging flow cytometry system. Similarly, the problem of “2D projection of 3D cell” also causes the false-negative signal, as this also causes the incidental overlap of the same fluorescent signals, and, again, this error could increase by examining more hybridization signals in spherical moving single cells [41]. In addition, a higher signal-to-noise ratio is required for the accurate detection of a positive signal in the imaging flow cytometry, while the detection of the real signal could be sometimes low with moving cells in solution. This also potentially causes the increase of false-negative results in the ISM-FISH. As one example of the relatively high rate of false-positive signals, we experienced false-positive signals of *IGH/FGFR3* in approximately 30% cells of tumor cells in KMS-21BM cells (Fig. 2b), while the false-positive rates were around 20% in patient-derived BM cells (Fig. 4). The most conceivable reasons for the high false-positive rate of *IGH/FGFR3* signal in KMS-21BM cells were the gene amplifications of *IGH* up to 6-8 copies, 3 copies of *MAF* and 4 copies of *FGFR3* in KMS-21BM cells as identified by DC-FISH (Supplementary Fig. 1). Indeed, the false-positive fusion signals for *IGH/FGFR3* and *IGH/MAF* were also detected in a few KMS-21BM cells with DC-FISH (data not shown). Such cytogenetic characteristics of KMS-21BM cells caused the increase of false-positive fusion signals of IGH/FGFR3 and IGH/MAF. However, such a huge gene amplification like *IGH* up to 6–8 copies is uncommon in patient-derived primary myeloma cells, and we were able to diagnose *IGH/FGFR3* by having an appropriate threshold even in cells with a high number of *IGH* amplification like KMS-21BM cells.

The other limitation is the lack of diagnostic ability of the current system in samples with deletion of der(14) containing the translocated *FGFR3* which occurs in up to 25% of t(4;14) patients [42], due to the use of probe for *FGFR3* for the t(4;14) detection instead of that for *NSD2*. However, the future change of probe that detects the *NSD2* gene may resolve this problem. Finally, due to the lack of ability for accurate signal quantification, the current ISM-FISH system is not suitable for the accurate evaluation of +1q and del(17p) in MM, and the accurate monitoring of tumor cell proportion in hematologic malignancies other than MM. Nevertheless, our ISM-FISH system provides a reliable qualitative diagnosis of three chromosomal translocations with the establishment of optimal thresholds simultaneously.

In conclusion, this study developed the new diagnostic system of ISM-FISH which enables the simultaneous diagnosis of three clinically pivotal chromosomal translocations, t(4;14), t(14;16), and t(11;14), in MGUS and MM. This system may facilitate rapid reliable cytogenetic diagnosis and promote patient-oriented therapy according to the type of chromosomal translocation in the setting of clinical practice.

## Supplementary information

Supplementary Figure 1

Supplementary Table 1

COMPETING INTERESTS

Supplementary Figure Legend
