# Supplementary figures and images for: Imaging flow cytometry-based multiplex FISH for three IGH translocations in multiple myeloma
Source: J Hum Genet. 2023 Mar 8;68(7):507–14. doi: 10.1038/s10038-023-01136-2 (PMC10290952; doi:10.1038/s10038-023-01136-2)

## Slide 1
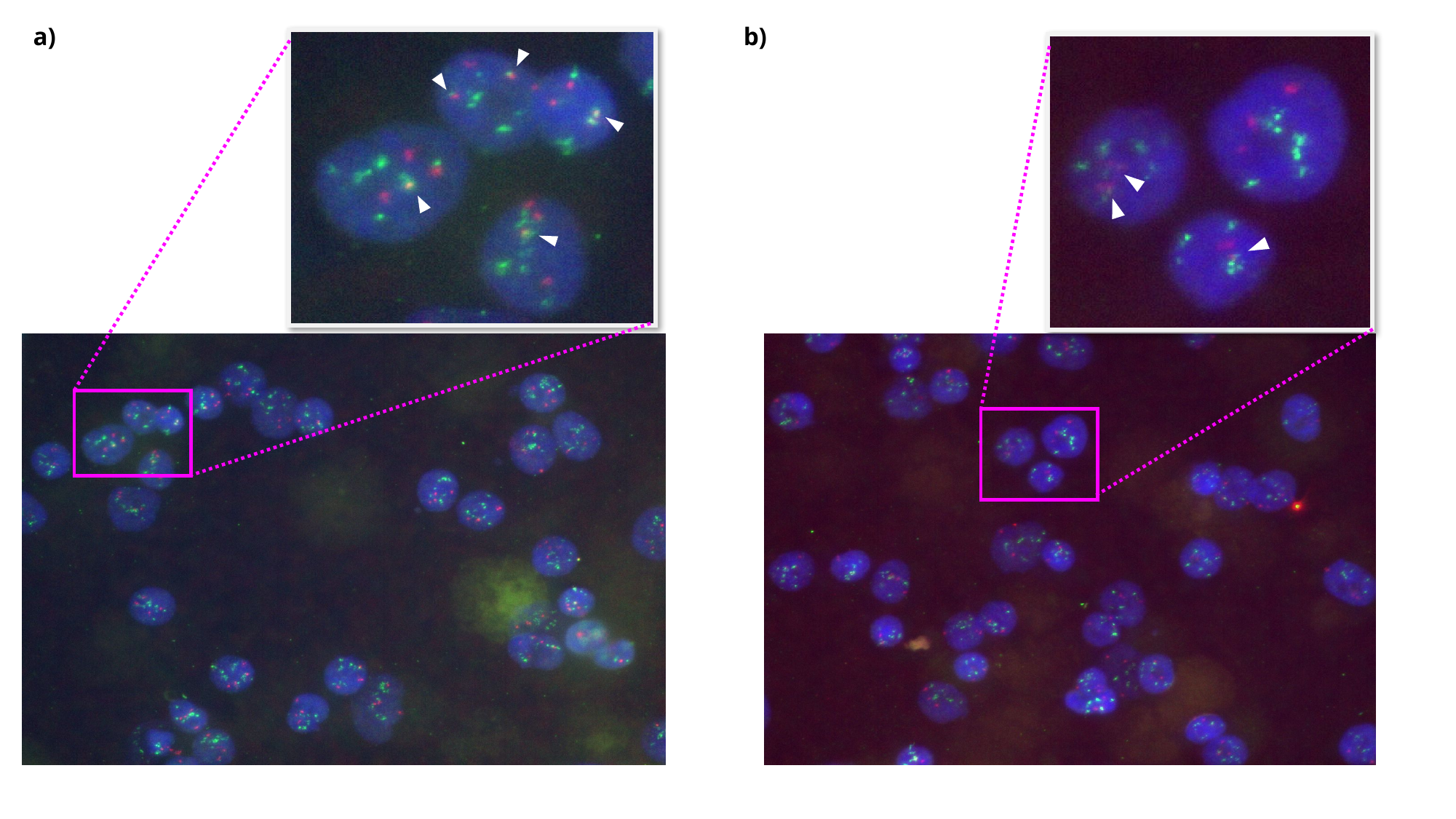

a)
b)

Supplement: Supplementary file 1 — Supplementary Figure 1 [file 10038_2023_1136_MOESM1_ESM.pptx]
