# Supplementary material for: Imaging flow cytometry-based multiplex FISH for three IGH translocations in multiple myeloma
Source: J Hum Genet. 2023 Mar 8;68(7):507–14. doi: 10.1038/s10038-023-01136-2 (PMC10290952; doi:10.1038/s10038-023-01136-2)
Supplement: Supplementary file 2 — Supplementary Table 1 [file 10038_2023_1136_MOESM2_ESM.docx]

| **Supplementary Table S1. Information of patient-derived samples** | | | | | | | |
| --- | --- | --- | --- | --- | --- | --- | --- |
| **No.** | **Dx.** | **BMPC (%)** | **Chromosomal translocation** | **Standard FISH with 200 BMNCs** | **ISM-FISH** | | |
|  |  |  |  | **positive cell ratio (%)** | **No. of total analyzed cells** | **No. of CD138-positive cells analyzed** | **Fusion signal-positive cells (%)** |
| 1 | RRMM | 63.6% | CCND1/IGH | 0.0% | 43822 | 2824 | 16.75% |
|  |  |  | FGFR3/IGH | 0.0% |  |  | 15.83% |
|  |  |  | MAF/IGH | 0.0% |  |  | 9.70% |
| 2 | RRMM | 43.6% | CCND1/IGH | 0.0% | 49789 | 2889 | 21.60% |
|  |  |  | FGFR3/IGH | 0.0% |  |  | 16.55% |
|  |  |  | MAF/IGH | 0.0% |  |  | 11.39% |
| 3 | RRMM | 14.2% | CCND1/IGH | 0.0% | 51901 | 425 | 23.29% |
|  |  |  | FGFR3/IGH | 0.0% |  |  | 16.94% |
|  |  |  | MAF/IGH | 0.0% |  |  | 9.18% |
| 4 | NDMM | 5.2% | CCND1/IGH | 3.0% | 63498 | 296 | 66.55% |
|  |  |  | FGFR3/IGH | 0.0% |  |  | 29.73% |
|  |  |  | MAF/IGH | 0.0% |  |  | 23.99% |
| 5 | RRMM | 2.0% | CCND1/IGH | 0.0% | 77077 | 127 | 20.47% |
|  |  |  | FGFR3/IGH | 0.0% |  |  | 18.90% |
|  |  |  | MAF/IGH | 0.0% |  |  | 8.66% |
| 6 | RRMM | 8.0% | CCND1/IGH | 0.0% | 91101 | 161 | 13.66% |
|  |  |  | FGFR3/IGH | 0.0% |  |  | 14.29% |
|  |  |  | MAF/IGH | 0.0% |  |  | 5.59% |
| 7 | NDMM | 10.2% | CCND1/IGH | 0.0% | 60032 | 74 | 28.38% |
|  |  |  | FGFR3/IGH | 0.0% |  |  | 18.92% |
|  |  |  | MAF/IGH | 0.0% |  |  | 14.86% |
| 8 | NDMM | 57.2% | CCND1/IGH | 5.0% | 79516 | 386 | 55.96% |
|  |  |  | FGFR3/IGH | 0.0% |  |  | 24.35% |
|  |  |  | MAF/IGH | 0.0% |  |  | 9.33% |
| 9 | NDMM | 35.0% | CCND1/IGH | 0.0% | 84486 | 415 | 15.90% |
|  |  |  | FGFR3/IGH | 0.0% |  |  | 26.27% |
|  |  |  | MAF/IGH | 0.0% |  |  | 11.08% |
| 10 | RRMM | 22.8% | CCND1/IGH | 0.0% | 53193 | 2292 | 19.55% |
|  |  |  | FGFR3/IGH | 0.0% |  |  | 17.28% |
|  |  |  | MAF/IGH | 0.0% |  |  | 6.54% |
| 11 | NDMM | 17.6% | CCND1/IGH | 0.0% | 55011 | 445 | 34.83% |
|  |  |  | FGFR3/IGH | 0.0% |  |  | 20.00% |
|  |  |  | MAF/IGH | 0.0% |  |  | 53.93% |
| 12 | NDMM | 58.2% | CCND1/IGH | 18.0% | 71394 | 1036 | 50.87% |
|  |  |  | FGFR3/IGH | 0.0% |  |  | 17.47% |
|  |  |  | MAF/IGH | 0.0% |  |  | 10.52% |
| 13 | NDMM | 53.4% | CCND1/IGH | 14.5% | 50740 | 17 | 52.94% |
|  |  |  | FGFR3/IGH | 0.0% |  |  | 35.29% |
|  |  |  | MAF/IGH | 0.0% |  |  | 23.53% |
| 14 | NDMM | 9.2% | CCND1/IGH | 5.0% | 71134 | 330 | 57.88% |
|  |  |  | FGFR3/IGH | 0.0% |  |  | 27.58% |
|  |  |  | MAF/IGH | 0.0% |  |  | 15.76% |
| 15 | NDMM | 31.0% | CCND1/IGH | 0.0% | 49094 | 194 | 23.20% |
|  |  |  | FGFR3/IGH | 0.0% |  |  | 24.23% |
|  |  |  | MAF/IGH | 0.0% |  |  | 12.89% |
| 16 | RRMM | 1.2% | CCND1/IGH | 0.0% | 59130 | 92 | 20.65% |
|  |  |  | FGFR3/IGH | 0.0% |  |  | 16.30% |
|  |  |  | MAF/IGH | 0.0% |  |  | 5.43% |
| 17 | RRMM | 1.6% | CCND1/IGH | 0.0% | 40481 | 32 | 28.13% |
|  |  |  | FGFR3/IGH | 0.0% |  |  | 25.00% |
|  |  |  | MAF/IGH | 0.0% |  |  | 15.63% |
| 18 | RRMM | 17.2% | CCND1/IGH | 0.0% | 59339 | 2385 | 24.53% |
|  |  |  | FGFR3/IGH | 14.0% |  |  | 66.29% |
|  |  |  | MAF/IGH | 0.0% |  |  | 15.01% |
| 19 | RRMM | 31.4% | CCND1/IGH | 0.0% | 53213 | 220 | 20.45% |
|  |  |  | FGFR3/IGH | 0.0% |  |  | 22.73% |
|  |  |  | MAF/IGH | 0.0% |  |  | 10.00% |
| 20 | RRMM | 47.6% | CCND1/IGH | 0.0% | 69784 | 3598 | 20.01% |
|  |  |  | FGFR3/IGH | 0.0% |  |  | 15.01% |
|  |  |  | MAF/IGH | 0.0% |  |  | 11.59% |
| 21 | RRMM | 8.4% | CCND1/IGH | 1.5% | 84615 | 244 | 75.41% |
|  |  |  | FGFR3/IGH | 0.0% |  |  | 27.05% |
|  |  |  | MAF/IGH | 0.0% |  |  | 12.70% |
| 22 | MGUS | 7.6% | CCND1/IGH | 0.0% | 84677 | 18 | 22.22% |
|  |  |  | FGFR3/IGH | 0.0% |  |  | 11.11% |
|  |  |  | MAF/IGH | 0.0% |  |  | 27.78% |
| 23 | RRMM | 26.8% | CCND1/IGH | 12.5% | 89360 | 795 | 63.65% |
|  |  |  | FGFR3/IGH | 0.0% |  |  | 24.28% |
|  |  |  | MAF/IGH | 0.0% |  |  | 10.82% |
| 24 | RRMM | 5.0% | CCND1/IGH | 1.2% | 106136 | 81 | 43.21% |
|  |  |  | FGFR3/IGH | 0.0% |  |  | 23.46% |
|  |  |  | MAF/IGH | 0.0% |  |  | 16.05% |
| 25 | RRMM | 9.2% | CCND1/IGH | 0.0% | 43041 | 153 | 31.37% |
|  |  |  | FGFR3/IGH | 0.0% |  |  | 28.10% |
|  |  |  | MAF/IGH | 0.0% |  |  | 15.03% |
| 26 | NDMM | 11.2% | CCND1/IGH | 0.0% | 31884 | 309 | 21.04% |
|  |  |  | FGFR3/IGH | 0.0% |  |  | 9.39% |
|  |  |  | MAF/IGH | 0.0% |  |  | 11.65% |
| 27 | RRMM | 1.6% | CCND1/IGH | 0.0% | 60832 | 47 | 17.02% |
|  |  |  | FGFR3/IGH | 0.0% |  |  | 21.28% |
|  |  |  | MAF/IGH | 0.0% |  |  | 6.38% |
| 28 | RRMM | 9.2% | CCND1/IGH | 12.5% | 68378 | 364 | 65.93% |
|  |  |  | FGFR3/IGH | 0.0% |  |  | 25.27% |
|  |  |  | MAF/IGH | 0.0% |  |  | 9.62% |
| 29 | MGUS | 6.8% | CCND1/IGH | 0.0% | 62179 | 344 | 13.95% |
|  |  |  | FGFR3/IGH | 0.0% |  |  | 18.02% |
|  |  |  | MAF/IGH | 0.0% |  |  | 14.53% |
| 30 | RRMM | 12.6% | CCND1/IGH | 0.0% | 71889 | 232 | 21.12% |
|  |  |  | FGFR3/IGH | 0.0% |  |  | 17.24% |
|  |  |  | MAF/IGH | 0.0% |  |  | 10.78% |
| 31 | RRMM | 18.8% | CCND1/IGH | 0.0% | 45822 | 78 | 10.26% |
|  |  |  | FGFR3/IGH | 0.0% |  |  | 21.79% |
|  |  |  | MAF/IGH | 0.0% |  |  | 2.56% |
| 32 | MGUS | 1.8% | CCND1/IGH | 0.0% | 61246 | 154 | 18.18% |
|  |  |  | FGFR3/IGH | 3.4% |  |  | 46.75% |
|  |  |  | MAF/IGH | 0.0% |  |  | 18.18% |
| 33 | RRMM | 2.0% | CCND1/IGH | 0.0% | 52377 | 44 | 15.91% |
|  |  |  | FGFR3/IGH | 0.0% |  |  | 45.45% |
|  |  |  | MAF/IGH | 0.0% |  |  | 9.09% |
| 34 | RRMM | 29.0% | CCND1/IGH | 27.5% | 96018 | 359 | 55.99% |
|  |  |  | FGFR3/IGH | 0.0% |  |  | 18.94% |
|  |  |  | MAF/IGH | 0.0% |  |  | 12.26% |
| 35 | MGUS | 3.2% | CCND1/IGH | 0.0% | 70671 | 103 | 22.33% |
|  |  |  | FGFR3/IGH | 0.0% |  |  | 23.30% |
|  |  |  | MAF/IGH | 0.0% |  |  | 22.33% |
| 36 | RRMM | 0.2% | CCND1/IGH | 0.0% | 57420 | 91 | 20.88% |
|  |  |  | FGFR3/IGH | 0.0% |  |  | 18.68% |
|  |  |  | MAF/IGH | 0.0% |  |  | 19.78% |
| 37 | NDMM | 52.6% | CCND1/IGH | 0.0% | 37435 | 1117 | 19.61% |
|  |  |  | FGFR3/IGH | 0.0% |  |  | 18.53% |
|  |  |  | MAF/IGH | 0.0% |  |  | 13.16% |
| 38 | MGUS | 1.6% | CCND1/IGH | 4.0% | 67744 | 276 | 64.49% |
|  |  |  | FGFR3/IGH | 0.0% |  |  | 24.28% |
|  |  |  | MAF/IGH | 0.0% |  |  | 24.64% |
| 39 | MGUS | 1.2% | CCND1/IGH | 0.0% | 69364 | 104 | 31.73% |
|  |  |  | FGFR3/IGH | 0.0% |  |  | 32.69% |
|  |  |  | MAF/IGH | 0.0% |  |  | 14.42% |
| 40 | MGUS | 2.0% | CCND1/IGH | 0.0% | 81646 | 141 | 20.57% |
|  |  |  | FGFR3/IGH | 0.0% |  |  | 21.99% |
|  |  |  | MAF/IGH | 0.0% |  |  | 14.89% |
| 41 | MGUS | 4.4% | CCND1/IGH | 2.7% | 35592 | 153 | 50.98% |
|  |  |  | FGFR3/IGH | 0.0% |  |  | 26.80% |
|  |  |  | MAF/IGH | 0.0% |  |  | 21.57% |
| 42 | RRMM | 18.6% | CCND1/IGH | 10.5% | 101425 | 2253 | 54.64% |
|  |  |  | FGFR3/IGH | 0.0% |  |  | 19.80% |
|  |  |  | MAF/IGH | 0.0% |  |  | 10.96% |
| 43 | MGUS | 3.0% | CCND1/IGH | 0.0% | 71961 | 19 | 47.37% |
|  |  |  | FGFR3/IGH | 0.0% |  |  | 31.58% |
|  |  |  | MAF/IGH | 0.0% |  |  | 26.32% |
| 44 | NDMM | 32.0% | CCND1/IGH | 0.0% | 50842 | 2374 | 17.82% |
|  |  |  | FGFR3/IGH | 0.0% |  |  | 23.59% |
|  |  |  | MAF/IGH | 0.0% |  |  | 7.54% |
| 45 | MGUS | 5.8% | CCND1/IGH | 0.0% | 47920 | 287 | 21.95% |
|  |  |  | FGFR3/IGH | 0.0% |  |  | 23.69% |
|  |  |  | MAF/IGH | 0.0% |  |  | 15.33% |
| 46 | RRMM | 33.8% | CCND1/IGH | 0.0% | 56172 | 1338 | 27.50% |
|  |  |  | FGFR3/IGH | 0.0% |  |  | 25.41% |
|  |  |  | MAF/IGH | 0.0% |  |  | 14.35% |
| 47 | NDMM | 17.4% | CCND1/IGH | 0.0% | 68135 | 197 | 22.34% |
|  |  |  | FGFR3/IGH | 18.5% |  |  | 53.30% |
|  |  |  | MAF/IGH | 0.0% |  |  | 20.30% |
| 48 | NDMM | 18.4% | CCND1/IGH | 11.0% | 53951 | 473 | 55.39% |
|  |  |  | FGFR3/IGH | 0.0% |  |  | 23.89% |
|  |  |  | MAF/IGH | 0.0% |  |  | 11.21% |
| 49 | RRMM | 27.6% | CCND1/IGH | 0.0% | 98540 | 523 | 18.16% |
|  |  |  | FGFR3/IGH | 0.0% |  |  | 23.14% |
|  |  |  | MAF/IGH | 5.0% |  |  | 70.36% |
| 50 | NDMM | 19.4% | CCND1/IGH | 0.0% | 47877 | 974 | 15.49% |
|  |  |  | FGFR3/IGH | 0.0% |  |  | 14.37% |
|  |  |  | MAF/IGH | 0.0% |  |  | 10.88% |
| 51 | RRMM | 0.4% | CCND1/IGH | 0.0% | 84659 | 12 | 8.33% |
|  |  |  | FGFR3/IGH | 0.0% |  |  | 8.33% |
|  |  |  | MAF/IGH | 0.0% |  |  | 8.33% |
| 52 | RRMM | 33.0% | CCND1/IGH | 0.0% | 49178 | 383 | 16.71% |
|  |  |  | FGFR3/IGH | 0.0% |  |  | 22.45% |
|  |  |  | MAF/IGH | 17.0% |  |  | 73.11% |
| 53 | NDMM | 16.6% | CCND1/IGH | 0.0% | 58836 | 279 | 21.15% |
|  |  |  | FGFR3/IGH | 0.0% |  |  | 18.64% |
|  |  |  | MAF/IGH | 0.0% |  |  | 11.11% |
| 54 | MGUS | 3.6% | CCND1/IGH | 0.0% | 55449 | 71 | 19.72% |
|  |  |  | FGFR3/IGH | 0.0% |  |  | 18.31% |
|  |  |  | MAF/IGH | 0.0% |  |  | 19.72% |
| 55 | RRMM | 3.6% | CCND1/IGH | 0.0% | 76003 | 229 | 13.97% |
|  |  |  | FGFR3/IGH | 0.0% |  |  | 14.41% |
|  |  |  | MAF/IGH | 0.0% |  |  | 6.11% |
| 56 | NDMM | 34.2% | CCND1/IGH | 18.0% | 89604 | 4479 | 65.77% |
|  |  |  | FGFR3/IGH | 0.0% |  |  | 20.45% |
|  |  |  | MAF/IGH | 0.0% |  |  | 12.06% |
| 57 | NDMM | 37.8% | CCND1/IGH | 0.0% | 51845 | 68 | 27.94% |
|  |  |  | FGFR3/IGH | 0.0% |  |  | 22.06% |
|  |  |  | MAF/IGH | 18.5% |  |  | 89.71% |
| 58 | MGUS | 0.0% | CCND1/IGH | 0.0% | 98428 | 22 | 31.82% |
|  |  |  | FGFR3/IGH | 0.0% |  |  | 36.36% |
|  |  |  | MAF/IGH | 0.0% |  |  | 22.73% |
| 59 | NDMM | 34.8% | CCND1/IGH | 13.0% | 23558 | 112 | 21.43% |
|  |  |  | FGFR3/IGH | N.A |  |  | 34.82% |
|  |  |  | MAF/IGH | N.A. |  |  | 21.43% |
| 60 | RRMM | 9.4% | CCND1/IGH | 0.0% | 62431 | 751 | 18.24% |
|  |  |  | FGFR3/IGH | 13.5% |  |  | 51.66% |
|  |  |  | MAF/IGH | 0.0% |  |  | 13.58% |
| 61 | NDMM | 77.4% | CCND1/IGH | 0.0% | 63679 | 1652 | 20.70% |
|  |  |  | FGFR3/IGH | 0.0% |  |  | 18.58% |
|  |  |  | MAF/IGH | 0.0% |  |  | 10.59% |
| 62 | RRMM | 13.8% | CCND1/IGH | 0.0% | 60117 | 687 | 23.44% |
|  |  |  | FGFR3/IGH | 71.0% |  |  | 72.49% |
|  |  |  | MAF/IGH | 0.0% |  |  | 14.70% |
| 63 | RRMM | 33.4% | CCND1/IGH | 0.0% | 74210 | 67 | 20.90% |
|  |  |  | FGFR3/IGH | 3.5% |  |  | 46.27% |
|  |  |  | MAF/IGH | 0.0% |  |  | 14.93% |
| 64 | RRMM | 33.4% | CCND1/IGH | 42.0% | 50648 | 710 | 64.65% |
|  |  |  | FGFR3/IGH | 0.0% |  |  | 23.94% |
|  |  |  | MAF/IGH | 0.0% |  |  | 11.13% |
| 65 | NDMM | 33.0% | CCND1/IGH | 52.0% | 55440 | 1382 | 58.10% |
|  |  |  | FGFR3/IGH | 0.0% |  |  | 24.96% |
|  |  |  | MAF/IGH | 0.0% |  |  | 26.85% |
| 66 | RRMM | 21.0% | CCND1/IGH | 0.0% | 146118 | 67 | 17.91% |
|  |  |  | FGFR3/IGH | 1.0% |  |  | 79.10% |
|  |  |  | MAF/IGH | 0.0% |  |  | 14.93% |
| 67 | NDMM | 63.4% | CCND1/IGH | 45.5% | 50462 | 269 | 45.35% |
|  |  |  | FGFR3/IGH | 0.0% |  |  | 24.54% |
|  |  |  | MAF/IGH | 0.0% |  |  | 11.90% |
| 68 | NDMM | 12.8% | CCND1/IGH | 0.0% | 133789 | 1023 | 19.75% |
|  |  |  | FGFR3/IGH | 0.0% |  |  | 20.82% |
|  |  |  | MAF/IGH | 0.0% |  |  | 14.08% |
| 69 | NDMM | 10.2% | CCND1/IGH | 0.0% | 79711 | 2329 | 17.90% |
|  |  |  | FGFR3/IGH | 13.5% |  |  | 70.30% |
|  |  |  | MAF/IGH | 0.0% |  |  | 12.80% |
| 70 | NDMM | 78.0% | CCND1/IGH | 0.0% | 91544 | 3928 | 18.51% |
|  |  |  | FGFR3/IGH | 49.0% |  |  | 74.92% |
|  |  |  | MAF/IGH | 0.0% |  |  | 12.75% |

Abbreviations. No.: number. Dx.: Diagnosis, BMPC: bone marrow plasma cell, BMNC: BM nucleated cell, NDMM: newly diagnosed multiple myeloma (MM), RRMM: refractory/relapsed MM, MGUS: monoclonal gammopathy of undermined significance, FISH: florescence in situ hybridization.
