# Supplementary material for: Imaging flow cytometry-based multiplex FISH for three IGH translocations in multiple myeloma
Source: J Hum Genet. 2023 Mar 8;68(7):507–14. doi: 10.1038/s10038-023-01136-2 (PMC10290952; doi:10.1038/s10038-023-01136-2)
Supplement: Supplementary file 4 — Supplementary Figure Legend [file 10038_2023_1136_MOESM4_ESM.docx]

**Figure S1.** Double-color FISH studies for **a.** *IIGH/FGFR3*, and **b.** *IGH/MAF* in KMS-21-BM cells*.* The green signal indicates IGH, while the red signals indicate *FGFR3* (**a**), and *MAF* (**b**). Arrows indicate false-positive fusion signals.
